# Growth-promoting effect of oestriol in a lymphoma lacking oestrogen receptors.

**DOI:** 10.1038/bjc.1989.114

**Published:** 1989-04

**Authors:** R. Kawatsu, T. Ezaki, M. Kotani, M. Akagi

**Affiliations:** 2nd Department of Surgery, Kumamoto University Medical School, Japan.

## Abstract

**Images:**


Br. J. Cancer (1989), 59, 563–568

© The Macmillan Press, Ltd., 1989

### Growth-promoting effect of oestriol in a lymphoma lacking oestrogen receptors

R. Kawatsu[1], T. Ezaki[2], M. Kotani[2] & M. Akagi[1]

[1]*2nd Department of Surgery and* [2]*Department of Anatomy, Kumamoto University Medical School, 2-1, Honjo 2-Chome, Kumamoto-City, Kumamoto 860, Japan.*

**Summary** Various doses (1 μg to 10 mg) of oestriol ($E_3$) were intraperitoneally injected into mice immediately after subcutaneous inoculation of an oestrogen receptor-negative lymphoma cell line (KE-5) established from a spontaneously developed AKR thymic lymphoma. The growth of KE-5 cells was markedly promoted by $E_3$ at the early stage of tumour growth. At this stage, 1 μg $E_3$ enhanced tumour growth significantly and the maximum effect was obtained with 1 mg $E_3$. Normal female mice showed a higher incidence and shorter latency than males. However, once tumours became palpable, the tumour growth rate appeared to be unaffected. Histological observations using Alcian blue and colloidal iron revealed a marked increase of hyaluronic acid in the subcutaneous connective tissue of the tumour-injection site within 3–5 days after intraperitoneal administration of 1 mg $E_3$. Biochemical analyses showed a rapid and marked increase in skin hyaluronic acid content to over 3 times the control levels ($0.25 \pm 0.10$ mg $g^{-1}$ skin) within 3 days of $E_3$ administration. Subcutaneous inoculation of KE-5 cells together with hyaluronic acid (0.2 mg) resulted in markedly enhanced tumour growth, particularly at the early stage. These results suggest that an increase in stromal hyaluronic acid content is the most likely mechanism responsible for the promoting effect of $E_3$ on KE-5 cells.

Oestrogens may be carcinogenic and promote tumour growth in several cancers (Noble, 1964). The most common explanations have centred on oestrogen receptor-mediated cytological changes in target tumour cells. For example, oestrogens stimulate tumour cells to synthesise specific proteins (Rochefort *et al.*, 1986) and to increase nuclear RNA polymerase activity (Clark & Peck, 1979). Rochefort *et al.* (1986) have further suggested that autocrine growth factors can be encoded by oncogenes and expressed at a higher level in transformed cells. The participation of oestrogen-induced hormones including prolactin (Noble *et al.*, 1980) and progesterone (Clark & Peck, 1979) has also been reported.

It is also conceivable that oestrogens promote tumour growth indirectly by modulating the *in situ* tumour environment rather than directly stimulating tumour cells to proliferate via oestrogen receptors. Changes in various cellular components of the tumour environment may be involved in enhancement of tumour growth. Oestrogens enhance the phagocytic activity of macrophages (Nicol *et al.*, 1964; Sljivic & Warr, 1973). Although host macrophages have been reported to be tumoricidal (Evans, 1973; Levy & Wheelock, 1974), possible involvement of macrophages in enhancing the progression of certain tumours has also been demonstrated (Gorelik *et al.*, 1982; Mukai *et al.*, 1987). Oestrogens also stimulate mast cells (Asboe–Hansen, 1963): tumour-enhancing activity of mast cells has been reported (Roche, 1985), although these cells have also been characterized as antitumour effector cells (Farram *et al.*, 1980; Henderson *et al.*, 1981). Eosinophils contain oestrogen receptors (Tchernitchin & Tchernitchin, 1976), and anti-tumour activity of eosinophils (Jong & Klebanoff, 1980; Iwasaki *et al.*, 1986) has been demonstrated. Moreover, oestrogens have been reported to inhibit natural killer cell activity (Seaman *et al.*, 1978), and cell-mediated immunity (Waltman *et al.*, 1971; Luster *et al.*, 1980).

On the other hand, changes in the non-cellular components of the tumour environment may also be related to enhancement of tumour growth. Oestrogens induce an increase in the tissue contents of water and acid glycosaminoglycans, particularly hyaluronic acid in skin (Asboe-Hansen, 1963; Sobel *et al.*, 1965; Bentley *et al.*, 1986). Furthermore, the promoting effect of hyaluronic acid on tumour growth has been reported by many investigators, such as Takeuchi (1966) in Ehrlich ascites tumour cells and by Toole *et al.* (1979) in rabbit $V_2$ carcinoma.

In the present study, we found for the first time that the growth of an established thymic lymphoma having no oestrogen receptors was dramatically promoted by $E_3$. In order to clarify the possible mechanism of the promoting effect of $E_3$ on receptor-negative tumours, changes in the *in situ* tumour environment were studied in cellular and non-cellular components of subcutaneous connective tissue into which tumour cells were inoculated.

### Materials and methods

### *Animals*

Inbred AKR/Ms mice were originally obtained from Saitama Prefectural Cancer Centre (Saitama, Japan) and have been maintained in our laboratory by sib-mating for about 4 years. All were housed in a climate-controlled room and were fed standard laboratory chows and water *ad libitum*. Unless otherwise stated, male mice aged between 2 and 4 months were used for this study in order to obviate or minimise any undesirable disturbance by endogenous oestrogens.

### *Establishment of a thymic lymphoma cell line (KE-5)*

Small pieces of a freshly excised lymphoma that had spontaneously developed in the thymus of a 7-month-old female AKR/Ms mouse were incubated and agitated in Hank's medium containing 0.7 mg ml$^{-1}$ collagenase (WAKO Pure Chemical Industries Ltd, Osaka, Japan) at 37°C for 30 min. The digested cell suspension was filtered through a stainless steel mesh and washed with PBS. The cells were maintained in RPMI-1640 culture medium (Nissui Pharm. Co. Ltd, Tokyo, Japan), supplemented with 10% heat-inactivated fetal calf serum (FCS), penicillin (100 U ml$^{-1}$), streptomycin (0.1 mg ml$^{-1}$), $5 \times 10^{-5}$ M 2-mercaptoethanol (2ME), $2 \times 10^{-3}$ M L-glutamine and $1 \times 10^{-3}$ M sodium pyruvate at 37°C in an atmosphere of 5% $CO_2$ in air. During the maintenance of these original thymic lymphoma cells, several sublines were cloned by limiting dilution (0.25 cells per well). One of the sublines (KE-5) was characterised by stable and relatively slow growth *in vivo*, and this line was used throughout the present study. By indirect immunofluorescence using rat monoclonal antibodies (Sera Lab. Ltd, Sussex, England) against mouse T lymphocyte antigens, KE-5 cells were characterised as: Thy-1, highly positive; Lyt-1

Correspondence: M. Kotani.
Received 28 September 1988, and in revised form, 22 November 1988.

and Lyt-2, clearly negative. However, we were unable to detect any L3T4 antigen under our present assay conditions.

*Oestrogen receptor assay*

The biochemical quantitative assay of oestrogen receptor was carried out using tritiated oestradiol, $E_2$ ([2,4,6,7-$^3$H]-$E_2$; New England Nuclear, Boston, USA) and tritiated oestriol, $E_3$ ([2,4,6,7-$^3$H]-$E_3$; New England Nuclear) according to the method of Nishimura *et al.* (1982). As a positive control, a homogenate of uteri from 10 AKR mice was used in each $E_2$ and $E_3$ receptor assay. The amounts of oestrogen receptors in uterus and KE-5 cells were calculated by Scatchard analysis.

*Tumour growth*

Cultured KE-5 cells were washed twice in PBS and the cell suspension ($10^5$ cells in 0.2 ml PBS) subcutaneously injected into the shaved right flank of mice at the level of the twelfth rib. Various doses (1 $\mu$g to 10 mg) of $E_3$ in 1.0 ml of aqueous suspension (Estriel, Mochida Pharm. Co., Tokyo, Japan) were injected intraperitoneally into at least 10 mice in each group immediately after the KE-5 cell inoculation. The control mice were given 1.0 ml of solvent (Mochida; 50 mg arabic gum and 1 mg polysolvent 80 in 100 ml isotonic saline) without $E_3$ in the same manner. The day when tumours became palpable was designated as the day of onset of the tumour. The size of each tumour was thereafter measured daily with callipers until the day of death of each animal and expressed as the mean diameter in millimetres.

*Histological observations*

KE-5 cells ($10^5$ cells in 0.2 ml PBS) were subcutaneously injected into the right flank of male mice, and this was followed by intraperitoneal administration of 1 mg $E_3$ (1 mg ml$^{-1}$ solvent) or solvent alone. As non-tumour cell controls for KE-5 cells, normal thymoocytes ($10^5$ cells in 0.2 ml PBS) from the thymus of 4-week-old mice were injected subcutaneously in the same manner. Controls without cells were also prepared by injection of an equal volume of PBS in the same way as above. Each group consisted of specimens obtained from at least five mice. A papule about 7 mm in diameter usually formed at the injection site for a short time. Three to five days after the injection, the skin at the injection site was excised.

For studies on cellular components, the excised skin specimens were frozen and sectioned at a thickness of 6 $\mu$m. Macrophages were stained according to the method of Leder (1967) for non-specific esterase, and that of Barka & Anderson (1962) for acid phosphatase. Eosinophils were stained by the method of Graham & Karnovsky (1966) for peroxidase. Mast cells were also stained with toluidine blue for glycosaminoglycans (Culling *et al.*, 1985). The number of these cells located in the subcutaneous loose connective tissue under the panniculus carnosus into which KE-5 cells were usually inoculated was counted (cells per unit area; $5.76 \times 10^{-8}$ m$^2$) using an optical square micrometer.

For studies on non-cellular components, the excised skin specimens were fixed in Carnoy's solution, embedded in paraffin and sectioned at a thickness of 6 $\mu$m. The sections were stained with Alcian blue at pH 2.5 and pH 1.0 according to the method of Lev & Spicer (1964) for acid glycosaminoglycans and also with colloidal iron according to the method of Mowry (1958). In addition, collagen fibres were stained by van Gieson's method (Berry, 1898).

*Hyaluronic acid assay*

The content of hyaluronic acid in skin was measured in 10 mice each that had received 1 mg $E_3$ or solvent only intraperitoneally. The skins were excised, defatted and homogenised in 0.1 M Tris-HCl buffer (pH 7.5). The samples were then heated for 10 min in boiling water to denature enzymes in the skin. The skin homogenates were digested with 1 mg of pronase (protease, type XIV, Sigma, St Louis, USA) per ml of each sample at 37°C for 24 h. The digestion was repeated once more under the same conditions. The digested samples were then placed in boiling water for 10 min to destroy the enzyme activity, and dialysed against distilled water, which was changed at least two times. Residues were removed by centrifugation at 80 000 *g* for 1 h at 4°C. Aliquots (100 $\mu$l) of the supernatants were then digested with 0.01 U of streptococcal hyaluronidase (Hyaluronidase SD, Seikagaku Kogyo Co. Ltd, Tokyo) in 20 $\mu$l of 0.1 M phosphate buffer (pH 6.2) for 100 min at 37°C. The amounts of terminal *N*-acetylglucosamine residues produced by the enzymatic digestion of hyaluronic acid were measured by the method of Reissig *et al.* (1955). The content of hyaluronic acid was determined from the values of a standard curve obtained by digesting various amounts of hyaluronic acid (human umbilical cord, grade I, Sigma) with the enzyme and expressed as weight per gram wet skin weight.

*Tumour growth with hyaluronic acid*

KE-5 cells ($10^5$ cells) suspended in 0.2 ml PBS containing 0.2 mg hyaluronic acid were injected subcutaneously into 14 mice as described above. The same number of control mice received KE-5 cells only without hyaluronic acid. The day when a tumour became palpable was designated as its day of onset and the size of the tumour was thereafter measured daily with callipers until the death of each animal, being expressed as the mean diameter in mm.

*Statistical analysis*

Statistical significance of differences in mean values was assessed using Student's *t* test; a *P* value less than 0.05 was considered significant.

### Results

*Oestrogen receptor concentration in KE-5 lymphoma cells*

Biochemical analyses revealed an undetectable amount of $E_3$ receptor of less than 10 fmol ml$^{-1}$ protein, which was the lowest value measurable in the assay of the cytosol and nuclear fractions from KE-5 cells, whereas a substantial amount of $E_3$ receptor was detected in uterine tissue (40.6 fmol mg$^{-1}$ protein in cytosol and 26.2 fmol mg$^{-1}$ protein in the nuclear fraction) under the same assay conditions as shown in Figure 1. Although the affinity of $E_2$ for oestrogen receptor is known to be about ten times higher than that of $E_3$ (Martucci & Fishman, 1976), $E_2$ receptor was also undetectable in KE-5 cells, in contrast to much higher amounts of $E_2$ receptor in uterine tissue (431.4 fmol mg$^{-1}$ protein in cytosol and 234.8 fmol mg$^{-1}$ protein in the nuclear fraction) than those of $E_3$ receptor.

**Figure 1** Quantification of oestrogen receptor in uterus and KE-5 cells. Specific binding of $^3$H-oestrogens (E) by cytosol fractions or nuclear extracts (*y* axis) was plotted against the concentration of bound free $^3$H-E (*x* axis). ($E_2$ receptor, uterus: ○, KE-5 cells: ●; $E_3$ receptor, uterus: △, KE-5 cells: ▲.)

### *Effect of $E_3$ on KE-5 lymphoma cell growth*

Various doses (1 μg to 10 mg) of $E_3$ were intraperitoneally injected into mice immediately after subcutaneous inoculation of KE-5 cells ($10^5$ cells in 0.2 ml). The incidence and onset of tumours in mice are shown in Figure 2a. All groups of animals that received $E_3$ showed earlier onset and higher incidence of tumour in comparison with the group of controls that received solvent only without $E_3$. A dose of 1 μg $E_3$ enhanced tumour growth significantly. Tumours in mice that received 1 mg $E_3$ grew more rapidly and more frequently than those in mice that received 1–100 μg $E_3$ and showed a 100% incidence by 20 days after the tumour inoculation, whereas the maximum incidence in control mice was only about 60%. There was no difference in tumour incidence and onset between doses of 1 mg and 10 mg. Although markedly higher incidence and earlier onset of tumours in the early stage of tumour growth were observed in mice treated with $E_3$ than in solvent-administered controls, the growth rate after tumours had become palpable was not different between the two groups of mice treated with 1 mg $E_3$ and solvent only (Figure 2b).

**Figure 2** (**a**) Effect of various doses of $E_3$ on the incidence and onset of tumours in male mice. Either $E_3$ (1 μg: ▲, 10 μg: ■, 100 μg: ◆, 1 mg: ●, 10 mg: ★) or solvent (○) was administered immediately after inoculation of KE-5 cells. (**b**) Effect of 1 mg $E_3$ on the growth rate of tumours after onset (solvent, ○; $E_3$, ●). Bars represent standard deviations. *P* value for each time point is greater than 0.05. (**c**) Effect of 1 mg $E_3$ on the incidence and onset of tumours in male and female mice. (Male, solvent: ○, $E_3$: ●; Female, solvent: △, $E_3$: ▲.)

The effect of 1 mg $E_3$ on the growth of KE-5 cells was compared between male and female mice (Figure 2c). Control female mice showed a higher incidence and earlier onset of tumours than control males. However, 1 mg $E_3$ raised the incidence of tumours in males to almost 90% of that in females. The latent period before onset of tumours was shortened by $E_3$ administration in both sexes.

### *Histological changes in tumour environment after $E_3$ administration*

The changes in cellular components in the subcutaneous loose connective tissue at the sites of injection of PBS alone, normal thymocytes and KE-5 cells for both the $E_3$- and solvent-administered control groups after 5 days are shown in Table I. No changes were found in the number and distribution of macrophages and mast cells in all groups, regardless of $E_3$ administration. Furthermore, the staining intensity of both enzymatic activities of macrophages and glycosaminoglycans of mast cells also showed no change. In contrast, eosinophils increased significantly in both solvent-administered groups for normal thymocyte ($P<0.01$) and KE-5 cell ($P<0.02$) inoculation in comparison with those in the other solvent group for PBS injection, indicating an increase in eosinophils after injection of thymocytes or KE-5 cells only in the absence of $E_3$. The number of eosinophils in the site of subcutaneous injection of PBS only in mice that had received intraperitoneal solvent only was not significantly different ($P>0.05$) from that in the subcutaneous connective tissue of non-treated normal controls. Comparing each of the groups, $E_3$ caused a marked decrease in the number of eosinophils in the groups administered PBS ($P<0.01$), thymocytes ($P<0.001$) and KE-5 cells ($P<0.001$). Fibroblasts showed little apparent morphological change after $E_3$ administration, as observed by the staining methods employed in this study.

As for changes in non-cellular components, acid glycosaminoglycan was stained with Alcian blue and colloidal iron. The subcutaneous loose connective tissue in all groups of mice that had received 1 mg $E_3$ 3–5 days previously was much more deeply stained with Alcian blue at pH 2.5 than that of the normal and solvent-administered control groups, as shown in Figure 3a and b. Colloidal iron staining also showed similar patterns of increased acid glycosaminoglycan. Such an increase in the staining intensity of this matrix was no longer detected when the samples were stained with Alcian blue at pH 1.0, indicating that the increased acid glycosaminoglycan in the subcutaneous loose connective tissue was hyaluronic acid having no sulphuryl, rather than chondroitin sulphate (Lev & Spicer, 1964). In addition, by van Gieson staining, a more loose and irregular arrangement of collagen fibres in the subcutaneous connective tissue of mice treated with $E_3$ than that of non-treated mice was observed, as shown in Figure 4a and b.

### *Effect of $E_3$ on the hyaluronic acid content of the skin*

The hyaluronic acid content per g of wet skin after intraperitoneal injection with and without 1 mg $E_3$ is shown in Figure 5. Compared with the amount in normal controls ($0.25 \pm 0.10\ mg\ g^{-1}$ skin), the hyaluronic acid content increased rapidly by 2.8-fold ($0.70 \pm 0.17\ mg\ g^{-1}$ skin) as early as 1 day after $E_3$ administration. The hyaluronic acid content reached its peak ($0.89 \pm 0.17\ mg\ g^{-1}$ skin) on day 3. Thereafter, it decreased gradually and returned to the normal level by day 14. On the other hand, solvent did not cause any increase in hyaluronic acid.

### *Effect of hyaluronic acid on KE-5 lymphoma cell growth*

KE-5 cells ($10^5$) in 0.2 ml PBS containing 0.2 mg hyaluronic acid were subcutaneously injected and the tumour growth

**Table I** Changes in the number of macrophages, mast cells and eosinophils in the subcutaneous connective tissues

| Cellular components tested | Normal control | Inoculation groups[a] PBS Solvent | | Thymocytes Solvent | | KE-5 cells Solvent | |
|---|---|---|---|---|---|---|---|
| | | *Solvent* | $E_3$ | *Solvent* | $E_3$ | *Solvent* | $E_3$ |
| Macrophages | | | | | | | |
| I[b] | 47±13[d] | 56±15 | 52±9 | 45±9 | 45±4 | 44±12 | 46±9 |
| II[c] | 39±23 | 38±11 | 36±8 | 33±9 | 30±3 | 42±13 | 27±4 |
| Mast cells | 0.9±0.9 | 0.5±0.5 | 0.4±0.9 | 1.0±0.7 | 0.5±0.3 | 1.0±0.7 | 0.3±0.3 |
| Eosinophils | 7±6 | 12±7 | 4±3 | 22±5 | 3±1 | 20±5 | 2±1 |

[a]Details are described in the text. Data were obtained 5 days after the inoculations.
[b]Acid phosphatase-positive cells.
[c]Non-specific esterase-positive cells.
[d]Mean ± standard deviation (s.d.) in $5.76 \times 10^{-8} m^2$.

**Figure 3** The subcutaneous loose connective tissue under panniculus carnosus (P) stained with Alcian blue at pH 2.5, 3 days after intraperitoneal administration of solvent (**a**) or 1 mg $E_3$ (**b**). The connective tissue is much more deeply stained in (**b**) than in (**a**). ×800.

**Figure 4** The subcutaneous loose connective tissue under the panniculus carnosus (P) stained by van Gieson's method, 5 days after intraperitoneal administration of solvent (**a**) or 1 mg $E_3$ (**b**). The collagen fibres are more loosely arranged in (**b**) than in (**a**). ×800.

was observed (Figure 6). The mice that received KE-5 cells together with hyaluronic acid showed a higher incidence and earlier onset of tumours than controls that received KE-5 cells only. The former showed an incidence as high as 92.9% by 20 days after the inoculation of KE-5 cells together with hyaluronic acid. On the other hand, the maximum incidence of tumours in control mice given KE-5 cells only without hyaluronic acid was less than 50%.

### Discussion

Although $E_3$ is believed to be absent in rodents (Turner & Bagnara, 1976) and has a much weaker effect on the reproductive organs than other compounds (Clark *et al.*, 1977), $E_3$ has been employed as a unique steroid that has a broad action on various other cells and tissues of rodents (Thompson *et al.*, 1965; Kotani *et al.*, 1979; Fujii *et al.*, 1985). The present study demonstrated that the growth of receptor-negative KE-5 lymphoma cells was markedly pro-

**Figure 5** The content of hyaluronic acid in skin after intraperitoneal administration of solvent (○) or 1 mg $E_3$ (●) to each of 10 mice. Bars represent standard deviations.

**Figure 6** The effect of hyaluronic acid on the incidence and onset of tumours in male mice. KE-5 cells ($10^5$ cells) were suspended in either PBS only (○) or PBS containing $1 \, mg \, ml^{-1}$ hyaluronic acid (●), and injected into the skin in a total volume of 0.2 ml.

moted by $E_3$ at the early stage of tumour growth. A small dose of $1 \mu g$ $E_3$ enhanced tumour growth significantly and 1 mg $E_3$ produced the maximum effect. Furthermore, it was suggested that endogenous oestrogen would be effective for tumour growth, since normal females showed higher incidence and shorter latency than normal males. However, it should be noted that after onset the growth rate of tumours appeared to be unaffected by $E_3$, possibly indicating that the latent period before onset is critical in this tumour model. The latent period may reflect tumour cell survival rather than cell proliferation. If so, then the tumour-promoting effect of $E_3$ may be interpreted simply as an $E_3$-induced improvement in the 'take' rate of KE-5 cells.

Histological observations on non-cellular components of the tumour environment revealed a rapid increase of hyaluronic acid in the subcutaneous connective tissue within 3–5 days after $E_3$ administration. In parallel with the histological observations, a more than three-fold increase in the hyaluronic acid content of the skin 3 days after $E_3$ administration was detected by biochemical assay. These results suggest a possible role of hyaluronic acid in tumour promotion at the early stage of tumour growth. This possibility was supported by observations of tumour growth after inoculation of KE-5 cells together with hyaluronic acid. The growth of KE-5 cells was improved by simultaneous injection of hyaluronic acid, and a great difference in incidence and latent period was also seen in the early developmental stage. However, the mechanisms of tumour promotion by hyaluronic acid are unclear at present. Rogers (1961) has suggested that hyaluronic acid is involved in the control of water retention, rate of diffusion, lubrication, macroionic function and so on. Takeuchi (1966) has proposed that hyaluronic acid protects the surface of tumour cells and promotes exchange of tumour metabolites. A higher concentration of acid glycosaminoglycans in various neoplastic tissues in comparison with non-neoplastic tissues has been reported by many investigators (Fukatsu *et al.*, 1988).

According to Bentley *et al.* (1986), the effects of oestrogen on an increase in hyaluronic acid in skin are probably mediated through oestrogen receptors in dermal fibroblasts. The dermal fibroblasts also have testosterone receptors (Jung-Testas *et al.*, 1976). Furthermore, it is known that testosterone increases the amount of metachromatic ground substance, particularly hyaluronic acid in skin (Branwood, 1963). Therefore, like oestrogens, testosterone may also have potent tumour-promoting activity. In contrast, adrenal cortical steroids inhibit the synthesis and metabolism of acid glycosaminoglycans in skin (Branwood, 1963; Asboe-Hausen, 1963). However, the effect of progesterone on hyaluronic acid production is still not established.

As another histological change in non-cellular components, we observed rearrangement of collagen fibres in the subcutaneous connective tissue after $E_3$ administration. Looseness or disorder of collagen fibres, which are considered to be one of the natural defence mechanisms against tumour invasion (Van den Hooff, 1983), may conceivably enhance infiltration of tumour cells into surrounding tissues. Such changes in collagen fibres may simply be induced by a rapid increase of intercellular matrix fluids including hyaluronic acid, whereas a direct influence of oestrogens on collagen metabolism has been reported by Bentley *et al.* (1986) and Hosokawa *et al.* (1981).

Histological observation on cellular components of the tumour environment showed that $E_3$ caused a marked reduction in the number of eosinophils in subcutaneous connective tissue compared with controls treated with solvent only. A decrease of eosinophils with antitumour activity (Jong & Klebanoff, 1980; Iwasaki *et al.*, 1986) may enhance the growth of KE-5 cells. However, such a cellular response may have resulted from the $E_3$-induced suppression of eosinophil infiltration, probably due to an ordinary inflammatory reaction after mechanical injury plus cellular effects by normal thymocytes or KE-5 cells. Moreover, solvent itself had little causative effect on eosinophil infiltration in the skin. Macrophages and mast cells showed no change in number, distribution or staining intensity due to $E_3$. Therefore, these cells seem unlikely to be major candidates for the mediation of $E_3$ tumour-promoting activity. In spite of the marked increase in hyaluronic acid content, fibroblasts showed little apparent morphological change due to $E_3$ in this study, as also reported by Branwood (1963).

Other immune mechanisms may also be involved in the promotion of tumour growth. The involvement of tumour-specific immune mechanisms mediated by immune lymphocytes may not be relevant to the present tumour model, because the tumour-promoting effect of $E_3$ was exhibited in too short a time for this to have been the case. Furthermore, NK cells may also not be involved in this tumour model, because long-term exposure to oestrogens is required for the reduction of NK activity (Seaman *et al.*, 1978). At the early stage of tumour growth, however, we cannot fully exclude the possibility that $E_3$ inhibits the production of some antitumour factors in the tumour microenvironment, such as interferons (Seaman *et al.*, 1979; Gresser *et al.*, 1979; Uno *et al.*, 1985), tumour necrosis factor (Carswell *et al.*, 1975) or tumour degenerating factor (Tanaka *et al.*, 1985).

In conclusion, the present study suggests that increased hyaluronic acid in the tumour environment may play a key role in the tumour growth-promoting effect of $E_3$. This experimental model may help to explain the growth-promoting effect of oestrogens on tumours lacking oestrogen receptors as in the case of receptor-negative melanoma which had its growth enhanced by oestrogens (Zava & Goldhirsch, 1983).

We wish to express our thanks to Dr M. Kimura, Dr N. Shigaki, Dr J. Yamashita and Dr N. Fujino, 2nd Department of Surgery, Kumamoto University Medical School, for their invaluable encouragement throughout this study. We are grateful to Dr H. Shisa, Saitama Prefectural Cancer Centre, for his supply of the original inbred AKR/Ms strain. We also thank Mrs E. Kinoshita for excellent technical help.

### References

ASBOE-HANSEN, G. (1963). The hormonal control of connective tissue. In *Internal Review of Connective Tissue Research*, Hall, D.A. (ed) vol. 1, p. 29. Academic Press: London.

BARKA, T. & ANDERSON, P.J. (1962). Histochemical methods for acid phosphatase using hexazonium pararosanilin as coupler. *J. Histochem. Cytochem.*, **10**, 741.

BENTLEY, J.P., BRENNER, R.M., LINDSTEDT, A.D. and 4 others (1986). Increased hyaluronate and collagen biosynthesis and fibroblast estrogen receptors in macaque sex skin. *J. Invest. Dermatol.*, **87**, 668.

BERRY, J.M. (1898). A comparison of the phagocytic action of leucocytes in amphibia and mammals. *Trans. Am. Microscop. Soc.*, **19**, 95.

BRANWOOD, A.W. (1963). The fibroblast. In *Internal Review of Connective Tissue Research*, Hall, D.A. (ed) vol. 1, p. 1. Academic Press: London.

CARSWELL, E.A., OLD, L.J., KASSEL, R.L., GREEN, S., FIORE, N. & WILLIAMSON, B. (1975). An endotoxin-induced serum factor that causes necrosis of tumors. *Proc. Natl Acad. Sci. USA*, **72**, 3666.

CLARK, J.H. PASZKO, Z. & PECK, E.J.P. JR (1977). Nuclear binding and retention of the receptor estrogen complex: relation to the agonistic and antagonistic properties of estriol. *Endocrinology*, **100**, 91.

CLARK, J.H. & PECK, E.J. JR (1979). Female sex steroids: receptors and function. *Monogr. Endocrinol.*, **14**, 1.

CULLIN, C.F.A., ALLISON, R.T. & BARR, W.T. (1985). *Cellular Pathology Technique*. Butterworths: London.

EVANS, R. (1973). Macrophages and the tumour bearing host. *Br. J. Cancer*, **28**, suppl. 1, 19.

FARRAM, E. & NELSON, D.S. (1980). Mouse mast cells as anti-tumor effector cells. *Cell Immunol.*, **55**, 294.

FUJII, H., HAYAMA, T. & KOTANI, M. (1985). Stimulating effect of natural estrogens on proliferation of hepatocytes in adult mice. *Acta Anat.*, **121**, 174.

FUKATSU, T., SOBUE, M., NAGASAKA, N. and 4 others (1988). Immunohistochemical localization of chondroitin sulphate and dermatan sulphate proteoglycans in tumour tissues. *Br. J. Cancer*, **57**, 74.

GORELIK, E., WILTROUT, R.H., BRUNDA, M.J., HOLDEN, H.T., & HERBERMAN, R.B. (1982). Augmentation of metastasis formation by thioglycollate-elicited macrophages. *Int. J. Cancer*, **29**, 575.

GRAHAM, R.C. & KARNOVSKY, M.J. (1966). The early stages of absorption of injected horseradish peroxidase in the proximal tubules of mouse kidney: ultrastructural cytochemistry by a new technique. *J. Histochem. Cytochem.*, **14**, 291.

GRESSER, I., DE MAEYER-GUIGNARD, J., TOVEY, M.G. & DE MAEYER, E. (1979). Electrophoretically pure mouse interferon exerts multiple biologic effects. *Proc. Natl Acad. Sci. USA*, **76**, 5308.

HENDERSON, W.R., CHI, E.Y., JONG, E.C. & KLEBANOFF, S.J. (1981). Mast cell-mediated tumor-cell cytotoxicity. Role of the peroxidase system. *J. Exp. Med.*, **153**, 520.

HOSOKAWA, M., ISHII, M., INOUE, K., YAO, C.S. & TAKEDA, T. (1981). Estrogen induces different responses in dermal and lung fibroblasts: special reference to collagen. *Connect. Tissues Res.*, **9**, 115.

IWASAKI, K., TORISU, M. & FUJIMURA, T. (1986). Malignant tumor and eosinophils. I. Prognostic significance in gastric cancer. *Cancer*, **58**, 1321.

JONG, E.C. & KLEBANOFF, S.J. (1980). Eosinophil-mediated mammalian tumor cell cytotoxicity: role of the peroxidase system. *J. Immunol.*, **124**, 1949.

JUNG-TESTAS, I., BAYARD, F. & BAULIEU, E.E. (1976). Two sex steroid receptors in mouse fibroblasts in culture. *Nature*, **259**, 136.

KOTANI, M., FUJII, H., TSUCHIYA, K., MATSUNO, K., EKINO, S. & HARADA, S. (1979). Effects of estrogen on the lymphoid regeneration and immune response in irradiated and marrow-reconstituted mice. *Acta Anat.*, **105**, 298.

LEDER, L.D. (1967). *Der Blutmonocyt*. Springer: Berlin.

LEV, R. & SPICER, S.S. (1964). Specific staining of sulphate groups with Alcian blue at low pH. *J. Histochem. Cytochem.*, **12**, 309.

LEVY, M.H. & WHEELOCK, E.F. (1974). The role of macrophages in defense against neoplastic disease. *Adv. Cancer Res.*, **20**, 131.

LUSTER, M.I., BOORMAN, G.A., DEAN, J.H., LUEBKE, R.W. & LAWSON, L.D. (1980). The effect of adult exposure to diethylstilbestrol in the mouse: alternations in immunological functions. *J. Reticuloendothel. Soc.*, **28**, 561.

MARTUCCI, C. & FISHMAN, J. (1976). Uterine estrogen receptor binding of catecholestrogens and of estetrol (1, 3, 5 (10)-estratriene-3, 15 alpha, 16 alpha, 17 beta-tetrol). *Steroids*, **27**, 325.

MOWRY, R.W. (1958). Improved procedure for the staining of acidic polysaccharides by Müller's colloidal (hydrous) ferric oxide and its combination with the Feulgen and the periodic acid-Schiff reactions. *Lab. Invest.*, **7**, 566.

MUKAI, M., SHINKAI, K., TATEISHI, R., MORI, Y. & AKEDO, H. (1987). Macrophage potentiation of invasive capacity of rat ascites hepatoma cells. *Cancer Res.*, **47**, 2167.

NICOL, T., BILBEY, D.L.J., CHARLES, L.M., CARDINGLEY, J.L. & VERNON-ROBERTS, B. (1964). Oestrogen: the natural stimulation of body defence. *J. Endocrinol.*, **30**, 277.

NISHIMURA, R., KIMURA, M., TOKUNAGA, T. & AKAGI, M. (1982). Measurement of nuclear estrogen receptors by charcoal adsorption; relationships of cytoplasmic and nuclear estrogen receptors and progesterone receptors in human breast cancer. *Gann*, **73**, 713.

NOBLE, R.L. (1964). Tumors and hormones. In *The Hormones*, Pincus, G., Thimann, K. & Astwood, E.B. (eds) vol. 5, p. 559. Academic Press: New York.

NOBLE, R.L., BEER, C.T. & GOUT, P.W. (1980). Evidence *in vivo* and *in vitro* of a role for the pituitary in the growth of malignant lymphomas in Nb rats. *Cancer Res.*, **40**, 2437.

REISSIG, J.L., STROMINGER, J.L. & LELOIR, L.F. (1955). A modified colorimetric method for the estimation of N-acetylamino sugars. *J. Biol. Chem.*, **217**, 959.

ROCHE, W.R. (1985). Mast cells and tumors. The specific enhancement of tumor proliferation *in vitro*. *Am. J. Pathol.*, **119**, 57.

ROCHEFORT, H., CAPONY, F., GARCIA, M. & VIGNON, F. (1986). The 52K estrogen-regulated protein secreted by breast cancer cells and its clinical potential. *Ann. NY Acad. Sci.*, **464**, 190.

ROGERS, H.J. (1961). The biochemistry of mucopolysaccharides of connective tissue, In *Biochemical Society Symposia*, no. 20, p. 51. Cambridge University Press: Cambridge.

SEAMAN, W.E., BLACKMAN, M.A., GINDHART, T.D., ROUBINIAN, J.R., LOEB, J.M. & TALAL, N. (1978). $\beta$-estradiol reduces natural killer cells in mice. *J. Immunol.*, **121**, 2193.

SEAMAN, W.E., MERIGAN, T.C. & TALAL, N. (1979). Natural killing in estrogen-treated mice responds poorly to poly I·C despite normal stimulation of circulating interferon. *J. Immunol.*, **123**, 2903.

SLJIVIC, V.S. & WARR, G.W. (1973). Oestrogens and immunity. *Period. Biol.*, **75**, 231.

SOBEL, H., LEE, K.D. & HEWLETT, M.J. (1965). Effect of estrogen on acid glycosaminoglycans in skin of mice. *Biochim. Biophys. Acta*, **101**, 225.

TAKEUCHI, J. (1966). Growth-promoting effect of acid mucopolysaccharides on Ehrlich ascites tumor. *Cancer Res.*, **26**, 797.

TANAKA, A., MATSUOKA, H., UEMURA, H. and 4 others (1985). Production and characterization of tumor-degenerating factor. *J. Natl Cancer Inst.*, **74**, 575.

TCHERNITCHIN, A. & TCHERNITCHIN, X. (1976). Characterization of the estrogen receptors in the uterine and blood eosinophil leukocytes. *Experientia*, **32**, 1240.

THOMPSON, J.S., REILLY, R.W., CRAWFORD, M. & RUSSE, H.P. (1965). The effect of estradiol and estriol on the survival of sublethally and lethally irradiated mice. *Radiat. Res.*, **26**, 567.

TOOLE, B.P., BISWAS, C. & GROSS, J. (1979). Hyaluronate and invasiveness of the rabbit $V_2$ carcinoma. *Proc. Natl Acad. Sci. USA*, **76**, 6299.

TURNER, C.D. & BAGNARA, J.T. (1976). *General Endocrinology*, p. 456. Saunders: Philadelphia.

UNO, K., SHIMIZU, S., IDO, M. and 5 others (1985). Direct and indirect effects of interferon on *in vivo* murine tumor cell growth. *Cancer Res.*, **45**, 1320.

VAN DEN HOOFF, A. (1983). Connective tissue changes in cancer. In *Internal Review of Connective Tissue Research*, Hall, D.A. & Jackson, D.S. (eds) vol. 10, p. 395. Academic Press: London.

WALTMAN, S.R., BURDE, R.M. & BERRIOS, J. (1971). Prevention of corneal homograft rejection by estrogens. *Transplantation*, **11**, 194.

ZAVA, D.T. & GOLDHIRSCH, A. (1983). Estrogen receptor in malignant melanoma: fact or artefact? *Eur. J. Cancer Clin. Oncol.*, **19**, 1151.


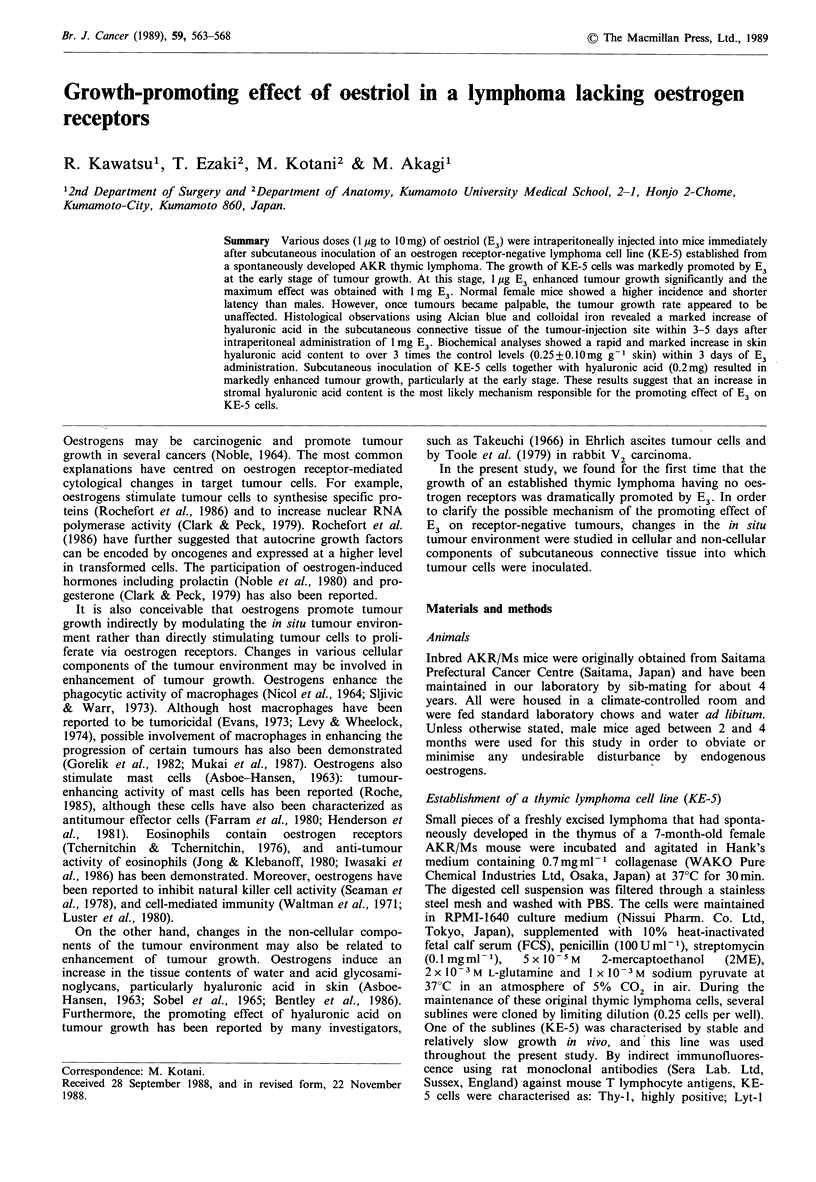

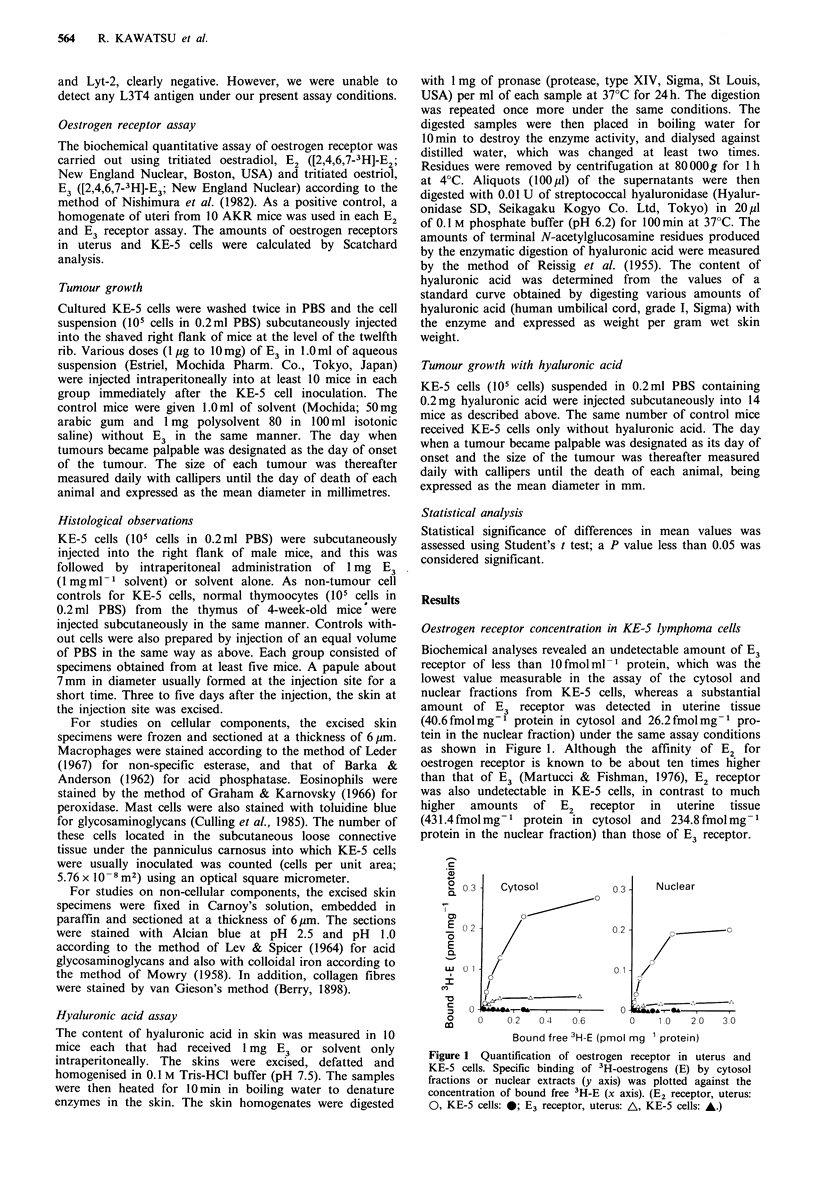

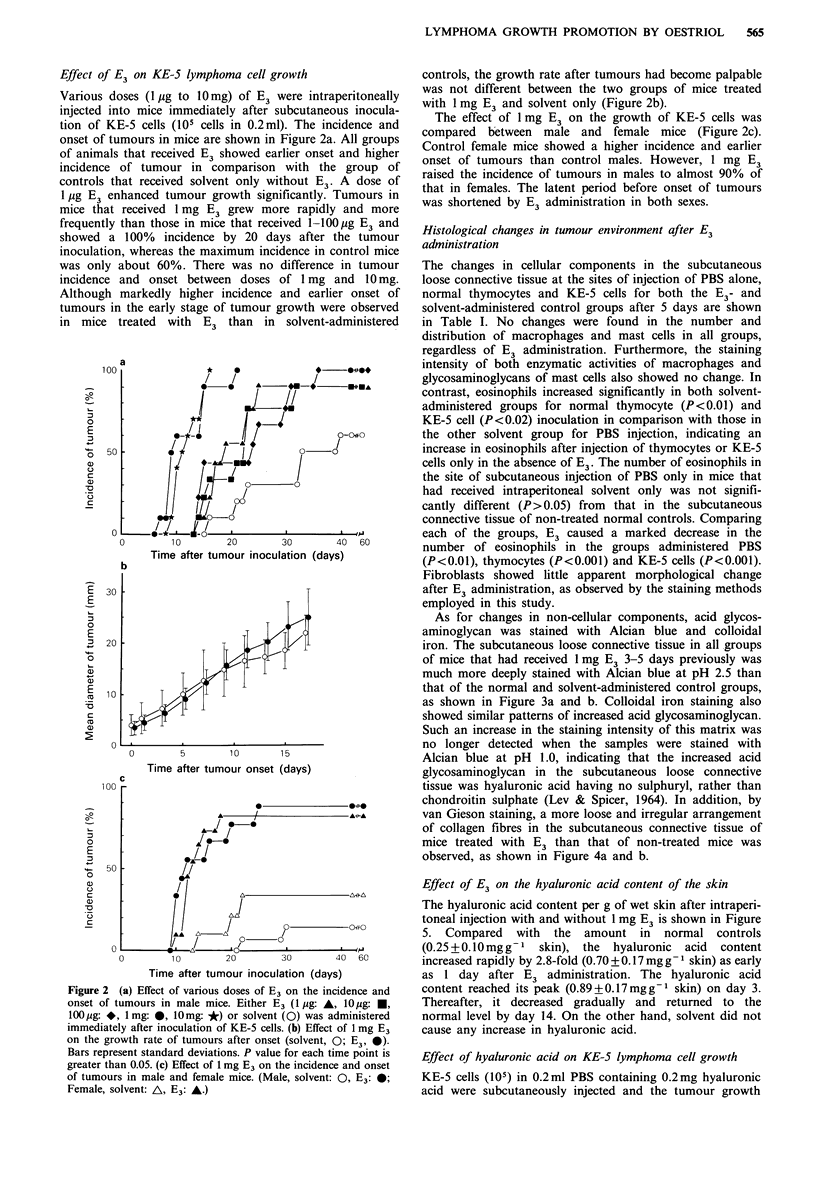

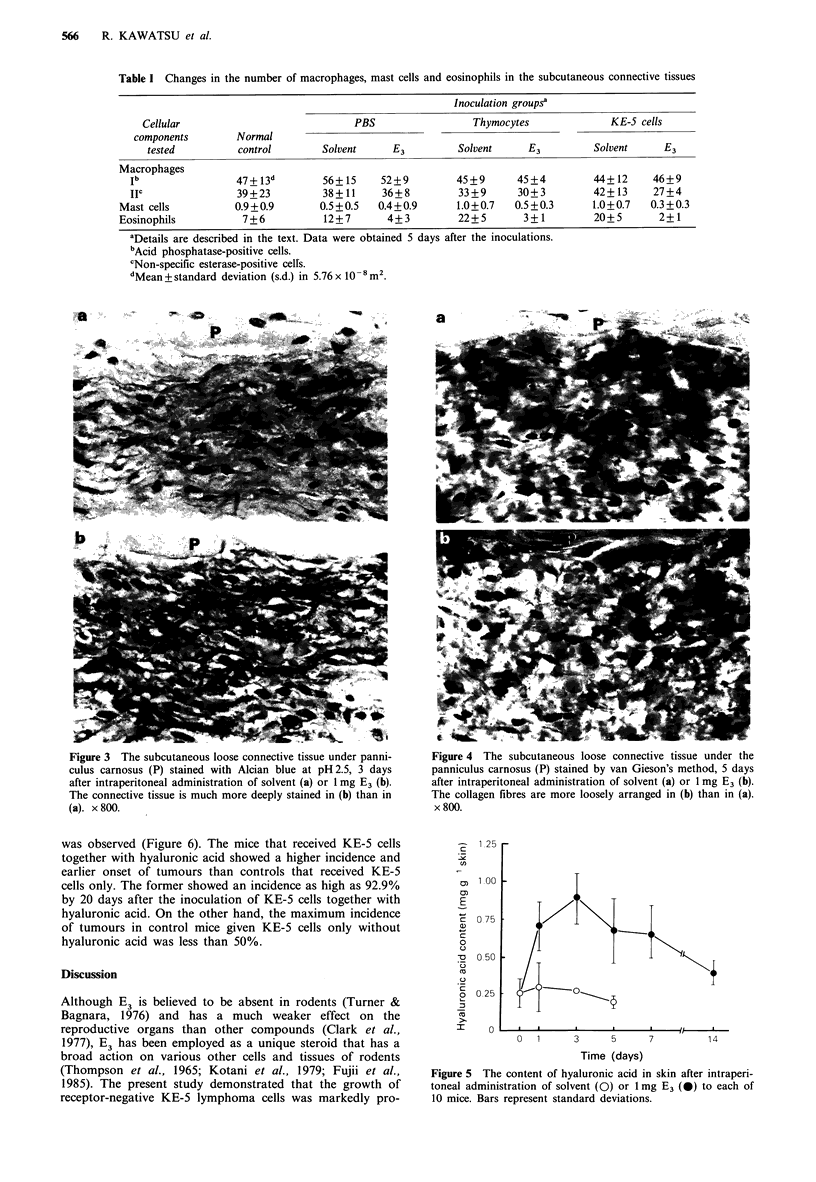

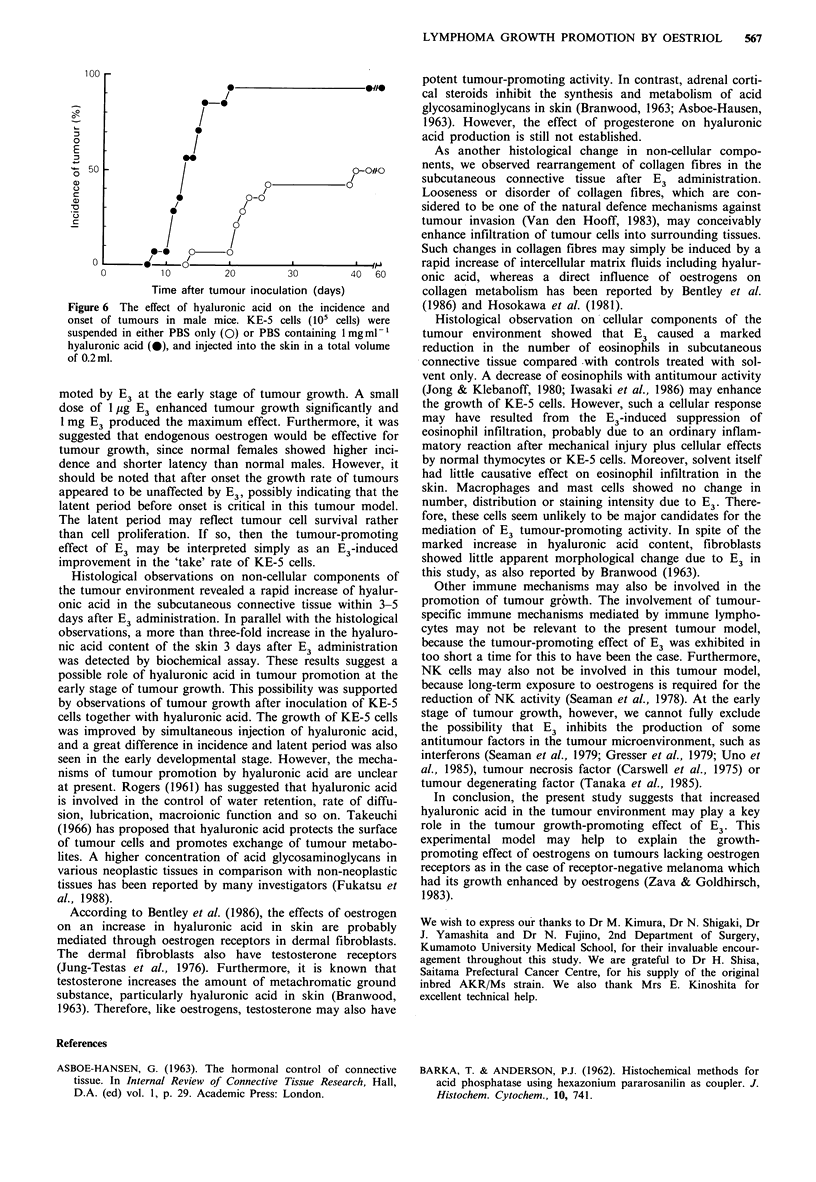

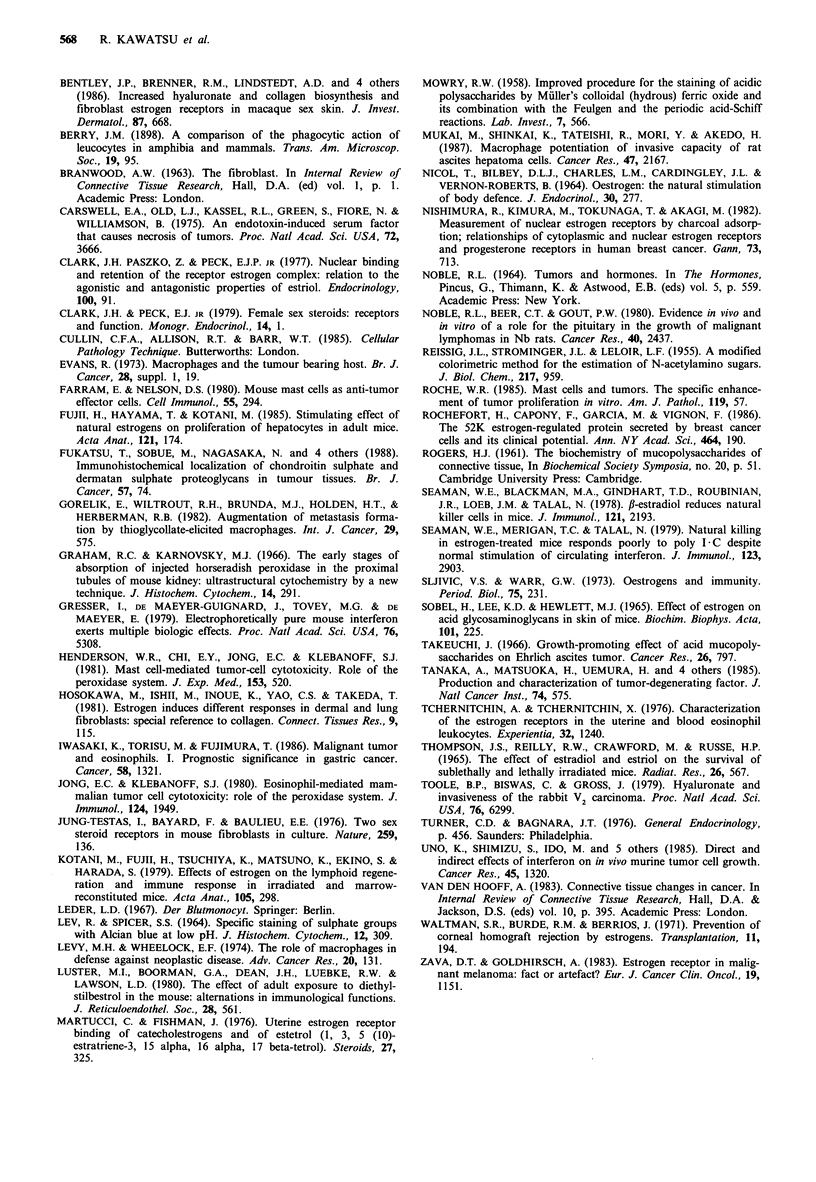


## References

[OCR_00800] Bentley J. P., Brenner R. M., Linstedt A. D., West N. B., Carlisle K. S., Rokosova B. C., MacDonald N. (1986). Increased hyaluronate and collagen biosynthesis and fibroblast estrogen receptors in macaque sex skin.. J Invest Dermatol.

[OCR_00816] Carswell E. A., Old L. J., Kassel R. L., Green S., Fiore N., Williamson B. (1975). An endotoxin-induced serum factor that causes necrosis of tumors.. Proc Natl Acad Sci U S A.

[OCR_00828] Clark J. H., Peck E. J. (1979). Female sex steroids: receptors and function.. Monogr Endocrinol.

[OCR_00840] Farram E., Nelson D. S. (1980). Mouse mast cells as anti-tumor effector cells.. Cell Immunol.

[OCR_00844] Fujii H., Hayama T., Kotani M. (1985). Stimulating effect of natural estrogens on proliferation of hepatocytes in adult mice.. Acta Anat (Basel).

[OCR_00855] Gorelik E., Wiltrout R. H., Brunda M. J., Holden H. T., Herberman R. B. (1982). Augmentation of metastasis formation by thioglycollate-elicited macrophages.. Int J Cancer.

[OCR_00861] Graham R. C., Karnovsky M. J. (1966). The early stages of absorption of injected horseradish peroxidase in the proximal tubules of mouse kidney: ultrastructural cytochemistry by a new technique.. J Histochem Cytochem.

[OCR_00867] Gresser I., De Maeyer-Guignard J., Tovey M. G., De Maeyer E. (1979). Electrophoretically pure mouse interferon exerts multiple biologic effects.. Proc Natl Acad Sci U S A.

[OCR_00873] Henderson W. R., Chi E. Y., Jong E. C., Klebanoff S. J. (1981). Mast cell-mediated tumor-cell cytotoxicity. Role of the peroxidase system.. J Exp Med.

[OCR_00878] Hosokawa M., Ishii M., Inoue K., Yao C. S., Takeda T. (1981). Estrogen induces different responses in dermal and lung fibroblasts: special reference to collagen.. Connect Tissue Res.

[OCR_00884] Iwasaki K., Torisu M., Fujimura T. (1986). Malignant tumor and eosinophils. I. Prognostic significance in gastric cancer.. Cancer.

[OCR_00889] Jong E. C., Klebanoff S. J. (1980). Eosinophil-mediated mammalian tumor cell cytotoxicity: role of the peroxidase system.. J Immunol.

[OCR_00894] Jung-Testas I., Bavard F., Baulieu E. E. (1976). Two sex steriod receptors in mouse fibroblasts in culture.. Nature.

[OCR_00899] Kotani M., Fujii H., Tsuchiya H., Matsuno K., Ekino S., Harada S. (1979). Effects of estrogen on the lymphoid regeneration and immune response in irradiated and marrow-reconstituted mice.. Acta Anat (Basel).

[OCR_00907] LEV R., SPICER S. S. (1964). SPECIFIC STAINING OF SULPHATE GROUPS WITH ALCIAN BLUE AT LOW PH.. J Histochem Cytochem.

[OCR_00911] Levy M. H., Wheelock E. F. (1974). The role of macrophages in defense against neoplastic disease.. Adv Cancer Res.

[OCR_00915] Luster M. I., Boorman G. A., Dean J. H., Luebke R. W., Lawson L. D. (1980). The effect of adult exposure to diethylstilbestrol in the mouse: alterations in immunological functions.. J Reticuloendothel Soc.

[OCR_00927] MOWRY R. W. (1958). Improved procedure for the staining of acidic polysaccharides by Müller's colloidal (hydrous) ferric oxide and its combination with the Feulgen and the periodic acid-Schiff reactions.. Lab Invest.

[OCR_00921] Martucci C., Fishman J. (1976). Uterine estrogen receptor binding of catecholestrogens and of estetrol (1,3,5(10)-estratriene-3,15alpha,16alpha,17beta-tetrol).. Steroids.

[OCR_00933] Mukai M., Shinkai K., Tateishi R., Mori Y., Akedo H. (1987). Macrophage potentiation of invasive capacity of rat ascites hepatoma cells.. Cancer Res.

[OCR_00938] NICOL T., BILBEY D. L., CHARLES L. M., CORDINGLEY J. L., VERNON-ROBERTS B. (1964). OESTROGEN: THE NATURAL STIMULANT OF BODY DEFENCE.. J Endocrinol.

[OCR_00943] Nishimura R., Kimura M., Tokunaga T., Akagi M. (1982). Measurement of nuclear estrogen receptors by charcoal adsorption: relationships of cytoplasmic and nuclear estrogen receptors and progesterone receptors in human breast cancer.. Gan.

[OCR_00955] Noble R. L., Beer C. T., Gout P. W. (1980). Evidence in vivo and in vitro of a role for the pituitary in the growth of malignant lymphomas in Nb rats.. Cancer Res.

[OCR_00960] REISSIG J. L., STORMINGER J. L., LELOIR L. F. (1955). A modified colorimetric method for the estimation of N-acetylamino sugars.. J Biol Chem.

[OCR_00965] Roche W. R. (1985). Mast cells and tumors. The specific enhancement of tumor proliferation in vitro.. Am J Pathol.

[OCR_00969] Rochefort H., Capony F., Garcia M., Vignon F. (1986). The 52K estrogen-regulated protein secreted by breast cancer cells and its clinical potential.. Ann N Y Acad Sci.

[OCR_00979] Seaman W. E., Blackman M. A., Gindhart T. D., Roubinian J. R., Loeb J. M., Talal N. (1978). beta-Estradiol reduces natural killer cells in mice.. J Immunol.

[OCR_00984] Seaman W. E., Merigan T. C., Talal N. (1979). Natural killing in estrogen-treated mice responds poorly to poly I.C despite normal stimulation of circulating interferon.. J Immunol.

[OCR_00994] Sobel H., Lee K. D., Hewlett M. J. (1965). Effect of estrogen on acid glycosaminoglycans in skin of mice.. Biochim Biophys Acta.

[OCR_00999] Takeuchi J. (1966). Growth-promoting effect of acid mucopolysaccharides on Ehrlich ascites tumor.. Cancer Res.

[OCR_01003] Tanaka A., Matsuoka H., Uemura H., Kakui Y., Imanishi T., Nishino H., Imanishi J. (1985). Production and characterization of tumor-degenerating factor.. J Natl Cancer Inst.

[OCR_01008] Tchernitchin A., Tchernitchin X. (1976). Characterization of the estrogen receptors in the uterine and blood eosinophil leukocytes.. Experientia.

[OCR_01013] Thompson J. S., Reilly R. W., Crawford M., Russe H. P. (1965). The effect of estradiol and estriol on the survival of sublethally and lethally irradiated mice.. Radiat Res.

[OCR_01018] Toole B. P., Biswas C., Gross J. (1979). Hyaluronate and invasiveness of the rabbit V2 carcinoma.. Proc Natl Acad Sci U S A.

[OCR_01027] Uno K., Shimizu S., Ido M., Naito K., Inaba K., Oku T., Kishida T., Muramatsu S. (1985). Direct and indirect effects of interferon on in vivo murine tumor cell growth.. Cancer Res.

[OCR_01037] Waltman S. R., Burde R. M., Berrios J. (1971). Prevention of corneal homograft rejection by estrogens.. Transplantation.

[OCR_01042] Zava D. T., Goldhirsch A. (1983). Estrogen receptor in malignant melanoma: fact or artefact?. Eur J Cancer Clin Oncol.

